# The Influence of Optimal Sleep Onset Time and Duration on Risk of Stroke: A Community-Based, Cross-Sectional Study

**DOI:** 10.3390/jcm14186529

**Published:** 2025-09-17

**Authors:** Junyi Ma, Yang Wang, Ji Zhang, Li Tang, Yupeng Zhang, Sai Wang, Xuelun Zou, Lei Chen, Ye Li, Yi Zeng, Duolao Wang, Le Zhang

**Affiliations:** 1 Department of Neurology, Xiangya Hospital, Central South University, Nanchang 330006, China; 218102090@csu.edu.cn (J.M.);; 2 Department of Integrated Traditional Chinese and Western Medicine, Institute of Integrative Medicine, Xiangya Hospital, Central South University, Changsha 410008, China; wangyang_xy87@csu.edu.cn; 3 Department of Neurology, Xiangya Hospital, Central South University, Changsha 410008, China; 228102005@csu.edu.cn (L.T.); 208102083@csu.edu.cn (Y.Z.); w_s_ai@csu.edu.cn (S.W.); 228101020@csu.edu.cn (X.Z.); 218102089@csu.edu.cn (L.C.); 228102099@csu.edu.cn (Y.L.); 4 Department of Geriatrics, Second Xiangya Hospital, Central South University, Changsha 410011, China; zengyi_xyneuro@csu.edu.cn; 5 Department of Clinical Sciences, Liverpool School of Tropical Medicine, Liverpool L3 5QA, UK; 6 Brain Health Center of Hunan Province, Changsha 410000, China; 7 Human Brain Disease Biological Resources Platform of Hunan Province, Changsha 410008, China; 8 National Clinical Research Center for Geriatric Disorders, Xiangya Hospital, Central South University, Changsha 410075, China; 9 Multi-Modal Monitoring Technology for Severe Cerebrovascular Disease of Human Engineering Research Center, Changsha 410008, China; 10 FuRong Laboratory, Changsha 410078, China

**Keywords:** sleep duration, sleep onset time, ischemic stroke, intracerebral hemorrhage, risk

## Abstract

**Background:** While sleep duration’s association with stroke is established, the combined influence of sleep onset time and duration on stroke subtypes remains inadequately explored. Since circadian biology links sleep onset timing to vascular risk via mechanisms operating independently of sleep duration, we quantified their joint contributions to the risk of stroke. **Methods:** In this population-based cross-sectional study, from 31 December 2021 to 31 March 2022, we recruited 8168 ischemic stroke cases, 3172 intracerebral hemorrhage cases, and 13,458 control participants across 152 survey centers in 28 counties in Hunan Province, China. Standardized computer-assisted interviews collected sleep parameters. Conjoint analysis identified protective sleep profiles. **Results:** Short sleep duration (<6 h) was consistently associated with elevated ischemic risk across all sleep onset times (*p* < 0.05 in all cases, i.e., sleep before 10 p.m. [odds ratio (95%CI): 1.886(1.606, 2.214)], 10–11 p.m. [1.740(1.336, 2.265)], 11 p.m.–12 a.m. [2.335(1.190, 4.581)], and after 12 a.m. [2.834(1.193, 6.728)]). A sleep duration of 6–8 h with a sleep onset time between 10 p.m. and 12 a.m. was associated with the lowest ischemic risk (*p* < 0.001 in all cases). Conversely, prolonged sleep (>8 h) with an early sleep onset time (<10 p.m.) increased ischemic risk (OR 1.194, 95% CI 1.090–1.308, *p* < 0.001), whereas a late sleep onset time (11 p.m.–12 a.m.) in long sleepers was protective (OR 0.580, 95% CI 0.352–0.956, *p* < 0.001). Similar trends were observed for ICH, though the effect sizes were attenuated. **Conclusion:** Sleep duration and onset time interact to influence stroke risk. Optimal cerebrovascular protection requires ≥6 h of sleep, ideally initiated between 10 p.m. and 11 p.m. These findings highlight sleep optimization as a potential modifiable target for high-risk populations.

## 1. Introduction

Sleep disturbances affect approximately 10–25% of the general population, with prevalence reaching 8.96–31.6% among Chinese adults [1,2,3], and nearly half of individuals aged ≥ 60 experience sleep initiation and maintenance difficulties [4]. Substantial evidence links sleep disturbances to cardiovascular [5] and cardiometabolic diseases [6], particularly stroke [7,8]. Both sleep duration and sleep onset timing are independently associated with cerebrovascular risk. Both short and long sleep durations demonstrate independent associations with elevated cerebrovascular risk [9,10,11], while habitual sleep exceeding 9 h correlates with hypertension and obesity-established precursors of stroke [12,13]. Notably, adults sleeping fewer than 6 h per night exhibit 20–32% greater hypertension risk compared to those maintaining 7–8 h [14]. In addition, previous studies have found that long shift work with disrupted circadian rhythms increases the risk of obesity, high blood pressure, diabetes, cardiovascular disease, and all-cause mortality [15,16,17,18,19]. Prolonged night shift exposure (>30 shifts, >15 consecutive nights, or >5 years) specifically increases cerebrovascular disease risk [20], likely through circadian disruption-induced activation of the inflammatory pathways involving TNFα and interleukins [21,22,23]. Although prior studies have identified associations between sleep disturbance and stroke risk, there has been less focus on the understanding of sleep patterns in the general population. Crucially, sleep onset time (SOT)—defined as the self-reported clock time at which an individual achieves persistent sleep each night—represents a distinct circadian marker whose interaction with duration remains unexamined in large-scale stroke studies. Current evidence remains constrained by a singular focus on isolated metrics, insufficient confounder adjustment, and limited sample diversity. There is a clear need for better evidence to guide healthier sleep patterns to reduce the risk of stroke.

This study quantifies the joint effects of sleep onset time and duration on stroke subtypes in 24,798 Hunan residents. Our design adjusts for key confounders while evaluating sleep pattern synergies. These cross-sectional analyses permit the assessment of associations but preclude causal inference.

## 2. Materials and Methods

### 2.1. Cross-Sectional Study Design and Participants

Our study is a large-scale cross-sectional investigation of sleep pattern associations (duration and onset time) with stroke occurrence, conducted among permanent residents of Hunan Province in Central China. Details of the study design and methodology have been reported previously [24]. In brief, trained investigators implemented multistage stratified cluster sampling across the province, carrying out the baseline survey between 31 December 2021 and 31 March 2022. All participants were recruited through structured community engagement utilizing primary healthcare infrastructure without advertisement-based approaches, with computer-assisted personal interviews administered at 152 pre-designated survey centers by certified investigators from township-level hospitals and village clinics.

**Figure 1:**
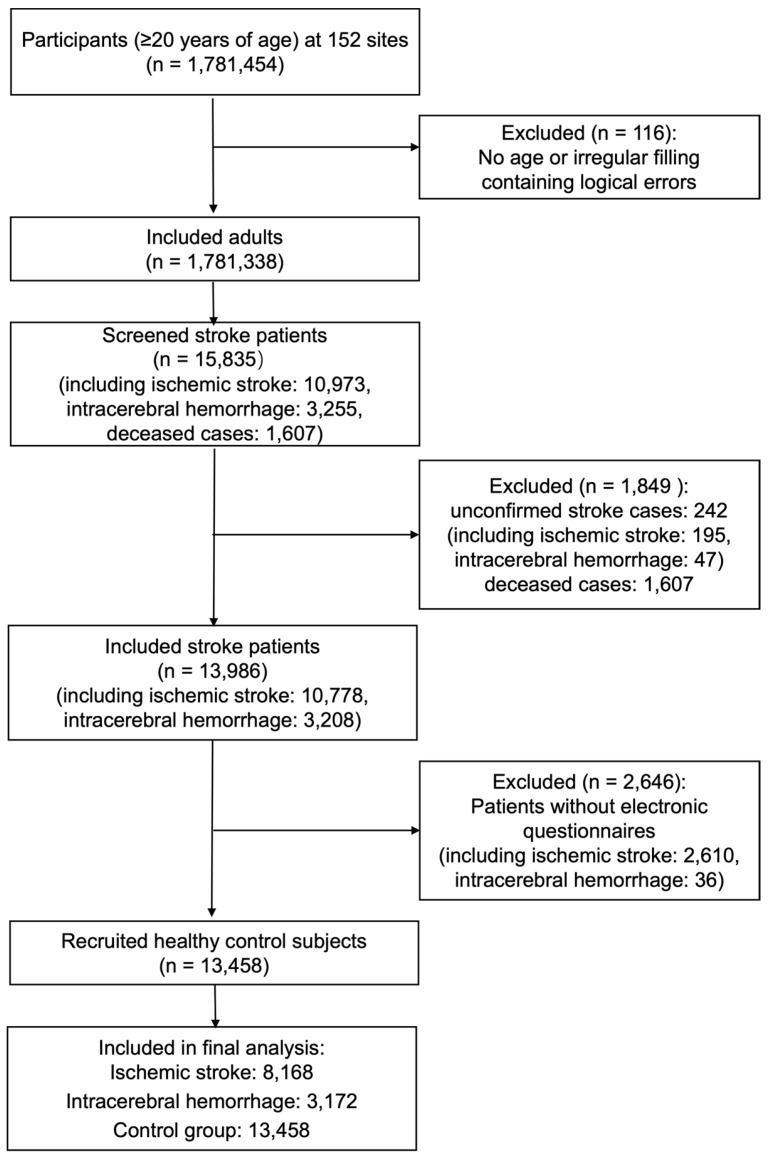
Evaluation flowchart of stroke cases in the epidemiologic investigation. A total of 1,781,454 participants aged ≥ 20 years were recruited from 152 sites in Hunan Province. After excluding 116 individuals with missing age or data errors, 1,781,338 adults were retained. Among these, 15,835 screened stroke cases were identified (including 10,973 ischemic strokes and 3255 intracerebral hemorrhages). Following the exclusion of 242 unconfirmed stroke cases (195 ischemic strokes and 47 intracerebral hemorrhages), 13,986 verified stroke cases remained (10,778 ischemic strokes; 3208 intracerebral hemorrhages). After further excluding 2646 patients lacking electronic questionnaires (2610 ischemic strokes; 36 intracerebral hemorrhages), 13,458 healthy controls were recruited. The final analysis included 8168 ischemic stroke cases, 3172 intracerebral hemorrhage cases, and 13,458 controls.

From 1,781,454 initially screened community residents in Hunan Province, we excluded 116 individuals due to missing age information or illogical data inconsistencies. The final analytical cohort comprised 1,781,338 adults aged ≥ 20 years. Stroke diagnoses were confirmed according to standardized criteria: neuroimaging evidence (CT/MRI reports), clinical documentation in medical records, official death certification or autopsy findings, and final validation by consulting neurologists. Within this cohort, 15,835 stroke cases were identified through screening (including 10,973 ischemic strokes [IS], 3255 intracerebral hemorrhages [ICHs], and 1607 deceased cases). After excluding 242 unconfirmed stroke cases (195 IS; 47 ICH), 13,986 verified stroke cases remained (10,778 IS; 3208 ICH). Among these, 11,340 patients (80.9%) completed electronic questionnaires (8168 IS; 3172 ICH). Additionally, 13,458 individuals without cardiovascular or cerebrovascular diseases from the same geographic area were enrolled as healthy controls (Figure 1).

### 2.2. Measurements

The main outcomes were the occurrences of IS and ICH at the time of the survey. The main exposure variables of sleep pattern were sleep duration and SOT. We used the data from the questions “How many hours have you slept most nights for the past six months?” and “What time have you fallen asleep most nights in the past six months?”, which provide estimates of sleep duration in seven categories and estimates of SOT points in five categories of participants (see Table S1). Based on the recommendation of a previous study, we divided the sleep duration into three categories: less than 6 h, 6–8 h, and more than 8 h. In addition, we divided SOT into four categories: sleep before 10 p.m., sleep between 10 and 11 p.m., sleep between 11 p.m. and 12 a.m., and sleep after 12 a.m.

We then included covariates of interest from the following two categories: (1) Participants’ basic demographic characteristics: age (year), gender (male, female), body mass index (calculating weight and height), marriage (married, unmarried, divorced, widowed), ethnic groups (Han nationality, other minorities), and education (*n* = 3 cohorts, none or lower than junior high school, junior high school qualification, senior high school qualification and higher). (2) Theoretical factors related to stroke, including smoking statues (smoker, non-smoker), alcohol abuse (alcohol abuser, non-alcohol abuser), snore (yes, no), history of coronary heart disease (yes, no, unknown), history of hyperlipidemia (yes, no, unknown), history of atrial fibrillation (yes, no, unknown), history of hypertension (yes, no, unknown), and history of diabetes (yes, no, unknown).

### 2.3. Data Analysis

Missing data patterns differed across the study groups. The ICH group exhibited missingness in ethnic groups (0.5%, *n* = 15), education (3.3%, *n* = 104), smoking status (3.0%, *n* = 95), and alcohol abuse (5.0%, *n* = 158). The IS group showed missingness in ethnic groups (0.5%, *n* = 43), education (3.5%, *n* = 283), smoking status (3.0%, *n* = 207), and alcohol abuse (3.9%, *n* = 316). The controls demonstrated missingness in ethnic groups (0.3%, *n* = 46), education (3.3%, *n* = 441), smoking status (2.0%, *n* = 269), and alcohol abuse (3.0%, *n* = 406). Sleep duration, SOT, diagnosis of stroke, and demographic variables (age, gender, BMI, marriage, snore, coronary heart disease, hyperlipidemia, atrial fibrillation, hypertension, and diabetes) were fully observed. Group-specific multivariate imputation by chained equations (MICE) addressed missingness under the missing-at-random assumption using R’s mice package (v3.15.0), generating 20 imputed datasets through 5 iterations per group. Random sampling addressed categorical variables (ethnicity, education, smoking status), incorporating all available predictors. Convergence was verified via trace plots, with final analyses using distinct imputed datasets (ICH: 9th; IS: 10th; controls: 8th). Validation regressions predicting hypertension history confirmed consistency between original and imputed data.

Associations between stroke occurrence and sleep pattern variables (sleep duration and SOT) were assessed using three hierarchically adjusted logistic regression models. Model 1 examined unadjusted associations. Model 2 incorporated core demographic covariates: age, sex, and body mass index (BMI). Model 3 adjusted for all covariates in Model 2 with additional confounders: educational level, marital status, ethnicity, smoking status, alcohol abuse, snore, and clinical histories of cardiovascular disease, hyperlipidemia, atrial fibrillation, hypertension, and diabetes. Covariate selection was guided by established epidemiological associations with sleep disorders or cerebrovascular outcomes. Conjoint analysis was employed to derive optimal pairings of sleep duration and SOT for IS and ICH.

Continuous variables are expressed as median with interquartile range (IQR), while categorical variables are reported as counts with percentages and as absolute and relative frequency for qualitative variables. Stratified analyses were conducted across subgroups defined by age (≤50 years, >50 years), sex (male, female), BMI (<24 kg/m^2^, ≥24 kg/m^2^), education (*n* = 3 cohorts, none or lower than junior high school, junior high school qualification, senior high school qualification and higher), smoking statues (smoker, non-smoker), and alcohol abuse (alcohol abuser, non-alcohol abuser). Furthermore, we conducted sensitivity analyses restricted to male participants to assess the robustness of our findings. Using identical modeling approaches as in the primary analysis, we evaluated the joint effects of sleep duration and onset time on stroke subtypes within this subgroup. All covariate adjustment strategies, imputation procedures, and statistical methods remained consistent with the main analyses.

All analyses were performed in R Studio (Version 2023.03.1+446) using the glmnet (v4.1-7), caret (v6.0-94), SparseM (v1.81), Hmisc (v5.1-0), Formula (v1.2-5), survival (v3.5-5), reportReg (v0.3.0), readr (v2.1.4), ISLR (v1.4), mice (v3.15.0), dplyr (v1.1.2), magrittr (v2.0.3), and CBCgrps (v1.0.1) packages. A significance threshold of *p* < 0.05 was used.

### 2.4. Language Editing

AI-assisted language editing was performed using DeepSeek-R1 (version R1, DeepSeek Co., Beijing, China) solely to enhance the readability and grammar of this paper. All scientific content remains generated by the authors.

## 3. Results

### 3.1. Baseline Characteristics

**Figure 2:**
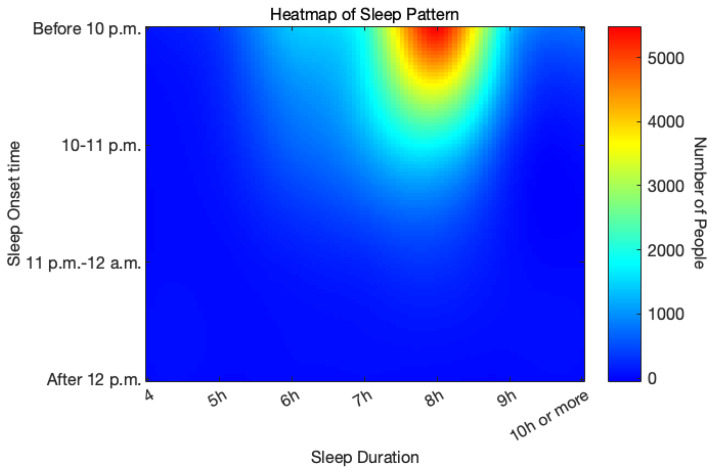
Heatmap of sleep onset time and sleep duration. The color bar represents the number of individuals in each sleep pattern, with red indicating high density and blue indicating low density.

We ultimately identified 8168 cases of IS, 3172 cases of hemorrhagic stroke, and 13,458 control participants. The baseline characteristics are presented in Table 1. Compared to the controls, both IS and ICH patients were significantly older, had a higher proportion of males, and a higher BMI. The majority of the IS and ICH patients were smokers and drinkers. Stroke patients exhibited significantly higher prevalence rates of hypertension, diabetes, atrial fibrillation, hyperlipidemia and cerebral vascular diseases, and snoring. Ethnicity distribution did not differ significantly between stroke patients and the controls. A heatmap visualizing sleep schedules (Figure 2) revealed that most participants initiated sleep before 10 p.m. and had a duration of around 8 h.

### 3.2. Sleep Onset Time and the Risk of Stroke

Logistic regression analyses demonstrated a significant association between SOT and the risk of both stroke types across all models. In unadjusted analyses related to SOT before 10 p.m., both SOT 10–11 p.m. (IS: OR = 0.623, 95% CI: 0.582–0.667, *p* < 0.001; ICH: OR = 0.637, 95% CI: 0.578–0.702, *p* < 0.001) and SOT 11 p.m.–12 a.m. (IS: OR = 0.443, 95% CI: 0.380–0.518, *p* < 0.001; ICH: OR = 0.567, 95% CI: 0.461–0.698, *p* < 0.001) were associated with reduced risk for both stroke types. SOT after 12 a.m. showed no significant risk alteration (IS: OR = 1.045, 95% CI: 0.756–1.444, *p* = 0.792; ICH: OR = 1.063, 95% CI: 0.680–1.661, *p* = 0.788). These associations remained significant even after adjusting for demographic, lifestyle, and clinical covariates (Models 2 and 3, Table 2).

### 3.3. Sleep Duration and the Risk of Stroke

In the unadjusted model, relative to a short sleep duration (<6 h), both intermediate (6–8 h) and long (>8 h) durations demonstrated protective associations. For the risk of IS, the unadjusted ORs were 0.475 (95% CI: 0.401–0.563, *p* < 0.001) for 6–8 h and 0.595 (95% CI: 0.493–0.718, *p* < 0.001) for >8 h. Similarly, for the risk of ICH, both intermediate (6–8 h) and long (>8 h) sleep durations showed protective associations, with unadjusted ORs of 0.475 (95% CI: 0.401–0.563; *p* < 0.001) and 0.595 (95% CI: 0.493–0.718; *p* < 0.001), respectively, compared to a short sleep duration (<6 h). Effect sizes were slightly attenuated modestly but retained statistical significance after adjusting for full covariate (Table 2).

### 3.4. Joint Effects of Sleep Onset and Duration

**Figure 3:**
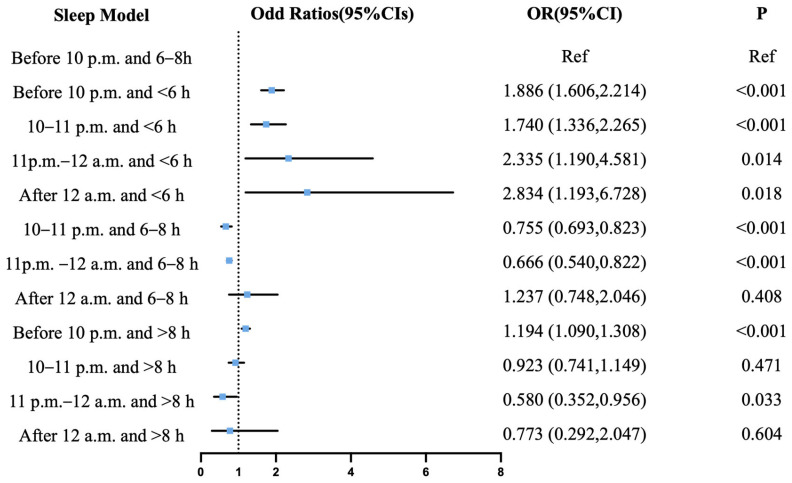
Joint effect of sleep onset time and sleep duration on ischemic stroke. Odds ratios are expressed for the risk of ischemic stroke for each sleep pattern compared to pattern of sleep before 10 p.m. with 6–8 h. CI = confidence interval; OR = odds ratio.

**Figure 4:**
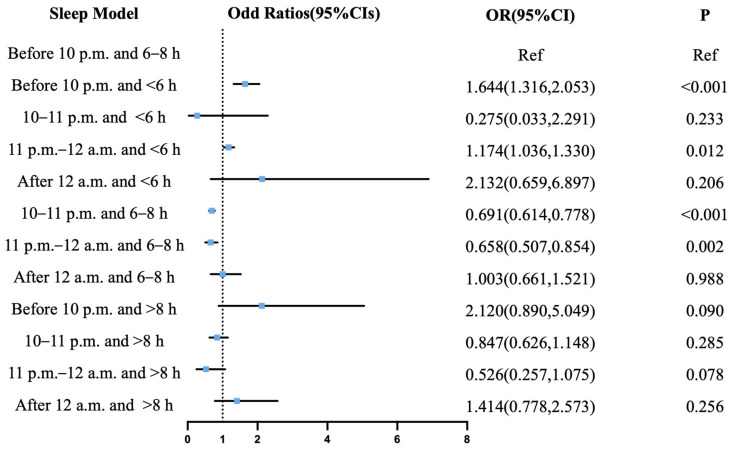
Joint effect of sleep onset time and sleep duration on intracerebral hemorrhage. Odds ratios are expressed for the risk of intracerebral hemorrhage for each sleep pattern compared to pattern of sleep before 10 p.m. with 6–8 h. CI = confidence interval; OR = odds ratio.

The comprehensive analysis of combined sleep onset time and duration patterns revealed distinct cerebrovascular risk profiles when using sleep initiation before 10 p.m. with a 6–8 h duration as the reference. For the risk of IS (Figure 3), protective associations were observed for sleep commencing between 10 and 11 p.m. with a 6–8 h duration (OR = 0.755, 95% CI: 0.693–0.823; *p* < 0.001), during 11 p.m.–12 a.m. with a 6–8 h duration (OR = 0.666, 95% CI: 0.540–0.822; *p* < 0.001), and during 11 p.m.–12 a.m. with a greater than 8 h duration (OR = 0.580, 95% CI: 0.352–0.956; *p* = 0.033). Conversely, a significantly elevated risk of IS corresponded to sleep initiation before 10 p.m. with a less than 6 h duration (OR = 1.886, 95% CI: 1.606–2.214; *p* < 0.001), between 10 and 11 p.m. with a less than 6 h duration (OR = 1.740, 95% CI: 1.336–2.265; *p* < 0.001), during 11 p.m.–12 a.m. with a less than 6 h duration (OR = 2.335, 95% CI: 1.190–4.581; *p* = 0.014), sleeping after 12 a.m. with a less than 6 h duration (OR = 2.834, 95% CI: 1.193–6.728; *p* = 0.018), and before 10 p.m. with a greater than 8 h duration (OR= 1.194, 95% CI: 1.090–1.308; *p* < 0.001). Regarding the risk of ICH (Figure 4), protective effects were observed for initiation between 10 and 11 p.m. combined with a 6–8 h duration (OR = 0.691, 95% CI: 0.614–0.778; *p* < 0.001) and during 11 p.m.–12 a.m. with a 6–8 h duration (OR= 0.658, 95% CI: 0.507–0.854; *p* = 0.002), whereas an elevated risk of ICH was associated with initiation before 10 p.m. with a less than 6 h duration (OR = 1.644, 95% CI: 1.316–2.053; *p* < 0.001) and during 11 p.m.–12 a.m. with a less than 6 h duration (OR = 1.174, 95% CI: 1.036–1.330; *p* = 0.012).

### 3.5. Subgroup Analysis

The observed inverse associations between delayed sleep onset time and extended sleep duration with reduced risk of stroke demonstrated consistent stability across all the examined demographic and behavioral strata. These associations remained robust when analyzed within subgroups stratified by age, sex, educational attainment, body mass index, smoking status, and alcohol consumption patterns, as visually documented in Figures S1–S4.

### 3.6. Sensitivity Analysis

Sensitivity analysis restricted to male participants yielded generally consistent patterns with the primary analysis for both stroke subtypes, though with notable variations in effect magnitude. For IS, a short sleep duration (<6 h) demonstrated significantly elevated risk across all sleep onset times, particularly for onset after 12 a.m. (OR = 9.49, 95% CI: 2.07–43.46). The protective effect of a 6–8 h sleep duration was most pronounced with onset at 11 p.m.–12 a.m. (OR = 0.69, 95% CI: 0.53–0.88), while long sleep (>8 h) with early onset (<10 p.m.) increased risk (OR = 1.20, 95% CI: 1.06–1.35) (Figure S5).

For ICH, males exhibited heightened vulnerability to a short sleep duration, especially with onset after 12 a.m. (OR = 9.68, 95% CI: 1.55–60.50). The 6–8 h sleep duration maintained protective associations when initiated at 10–11 p.m. (OR = 0.65, 95% CI: 0.56–0.75) or 11 p.m.–12 a.m. (OR = 0.69, 95% CI: 0.51–0.93). Unlike the primary analysis, no significant associations were observed between a long sleep duration and ICH risk in males (Figure S6).

## 4. Discussion

In this large cross-sectional analysis of over 24,798 participants, we examined associations between sleep patterns (SOT and sleep duration) and risk of stroke subtypes. A short sleep duration (<6 h) demonstrated significantly elevated cerebrovascular risk across all SOTs, with no observed risk attenuation through sleep timing adjustment. For individuals maintaining 6–8 h of sleep, cerebrovascular risk reduction was most pronounced when sleep initiation occurred between 10 p.m. and 12 a.m., whereas onset after 12 a.m. correlated with increased cerebrovascular risk.

Our findings revealed a U-shaped association between increased risk of stroke and SOT, with both early and very late SOT demonstrating elevated cerebrovascular risk. The present study shows that optimal SOT occurred between 11 p.m. and 12 a.m. This is consistent with the idea that optimal SOT falls within the range of the diurnal cycle, and points outside this range can be harmful. In healthy individuals, circadian regulation promotes peak wake-promoting signals around 19:00–22:00, and shortly after this peak, the release of melatonin promotes the rapid dissipation of arousal signals [25,26]. Delayed sleep initiation beyond this period induces circadian misalignment, which elevates cerebrovascular risk through hypertension induction and inflammatory cascade activation, evidenced by C-reactive protein elevation [27]. Furthermore, circadian dysregulation perturbs neuroendocrine rhythms involving leptin, glucose, insulin, and catecholamines [28]. Crucially, the nocturnal secretion nadir for these factors clusters between 22:30 and 23:00, suggesting that synchronizing sleep initiation within this interval may mitigate the risk of stroke by maintaining circadian homeostasis.

The present study suggested that 6–8 h of nightly sleep represents the optimal duration for reducing the risk of IS and ICH, whereas durations shorter than 6 h or longer than 8 h elevate cerebrovascular risk. These findings are generally consistent with prior epidemiological evidence demonstrating a U-shaped association between sleep duration and total stroke incidence, where the lowest risk is typically observed at 7 h of sleep [29,30]. This U-shaped pattern aligns with the results from large meta-analyses [30,31,32], including those summarized in recent reviews, which indicate that each hour of deviation from approximately 7 h of sleep significantly increases stroke risk [33]. Prospective cohort studies further substantiate this pattern, reporting an elevated risk of total stroke and IS with prolonged sleep, while abbreviated sleep duration is associated specifically with heightened cerebral hemorrhage susceptibility [34]. This may because a too short or long sleep duration is associated with some risk factors for stroke. A prospective study of 1268 hypertension patients found that those who reported sleep less than 7.5 h had a two-fold increased risk of stroke [35]. A large longitudinal study in China specifically focusing on individuals with metabolic syndrome found a significantly increased risk of incident stroke among those sleeping ≤ 6 h per night (HR 1.65; 95% CI 1.04–2.61) compared to 6–8 h, and similarly for sleep < 7 h/day (HR 1.62; 95% CI 1.03–2.53) compared to 7–9 h/day [31]. Biologically, sleep duration influences stroke pathogenesis through inflammatory or metabolic pathways. Sleep deprivation induces endothelial dysfunction and increased oxidative stress and inflammation [36] while concurrently activating sympathetic nerve activity and hypothalamic–pituitary–adrenal signaling to increase circulating cortisol [37,38]. Furthermore, inadequate sleep accelerates vascular structural deterioration, with acute sleep deprivation directly increasing arterial stiffness [39].

Various sleep patterns involving less than 6 h of sleep consistently demonstrated elevated risk of IS compared to longer-duration patterns, suggesting that extreme sleep deprivation may supersede circadian influences in cerebrovascular pathology. The established causal link between short sleep duration and vascular dysfunction is well-established, manifesting through direct endothelial impairment—including diminished flow-mediated dilation [40,41,42]—and indirect pathways such as disrupted glucose homeostasis and insulin resistance [43]. Critically, habitual short sleep may induce circadian misalignment, as desynchronization between behavior rhythms and physiological regulated functions elevates risks of aberrant blood pressure patterns [44,45]. A short sleep duration is associated with the unconventional circadian timing of feeding and activity, resulting in the misalignment of the master clocks in the brain with the peripheral clocks in organs [44,46]. While sleep duration interventions show promise for primary stroke prevention, differential optimal sleep patterns for IS risk versus ICH warrant mechanistic investigation, particularly regarding distinct cerebrovascular susceptibility to circadian disruption. Notably, we observed that a prolonged sleep duration (>8 h) combined with early sleep onset (<10 p.m.) significantly increased stroke risk, whereas long sleepers with late sleep onset (>12 a.m.) showed no elevated risk. This differential effect may be attributed to circadian biology: early sleep initiation during rising melatonin levels may extend the nocturnal melatonin peak, potentially disrupting the circadian regulation of cardiovascular functions. Conversely, late SOT in long sleepers may better align sleep architecture with core circadian processes, as melatonin secretion typically peaks around 2–4 a.m. [47], coinciding with the middle of the sleep period in these individuals.

### Strengths and Limitation

This study has several notable strengths. First, it represents the inaugural investigation into the combined effects of sleep onset timing and duration on the risk of stroke, providing novel evidence on sleep patterns rarely examined in prior research. Second, the large-scale provincial sampling across Hunan’s diverse geographical expanse (exceeding some countries in area) enhances population representativeness. Third, our multistage stratified cluster sampling strategy strengthened the external validity. Finally, extensive adjustment for sociodemographic, behavioral, and clinical confounders improved the robustness of the results.

However, this study’s limitations should be acknowledged. First, a cross-sectional design precludes causal inference between sleep patterns and stroke, necessitating longitudinal validation. Second, self-reported sleep measures introduce potential recall bias, and the exclusion of daytime napping metrics may mean that circadian influences are overlooked. Third, unmeasured confounding persists despite extensive covariate control, particularly regarding undiagnosed sleep apnea and genetic stroke predispositions. Fourth, selection bias is possible, as participation relied on voluntary survey responses, potentially underrepresenting individuals with severe health limitations or non-residents. Additionally, regional sampling within one Chinese province may limit extrapolation to populations with differing lifestyle patterns, though the sampling strategy mitigates this concern.

## 5. Conclusions

Our study suggests that sleep deprivation (<6 h) is associated with cerebrovascular risk independent of bedtime. The evidence indicates that maintaining 6–8 h of sleep between 10 p.m. and 11 p.m. may be beneficial for cerebrovascular health. Notably, individuals initiating sleep later (11 p.m.–12 a.m.) appeared to require a longer sleep duration (>8 h) to achieve comparable protective effects. These findings highlight the potential value of sleep timing and duration as modifiable factors and provide preliminary evidence to inform future research on personalized sleep interventions in cerebrovascular disease prevention.

## Figures and Tables

**Table 1:** Characteristics of intracerebral hemorrhage patients and controls.

Variable	Controls (*n* = 13,458)	Ischemic Stroke (*n* = 8168)	P_1_^a^	Intracerebral Hemorrhage (*n* = 3172)	P_2_^b^
Sex			<0.001		0.273
Female	5924 (44)	3354 (41)		1183 (37)	
Male	7534 (56)	4814 (59)		1989 (63)	
Age	67 (55, 74)	72 (65, 78)	<0.001	69 (59, 76)	<0.001
BMI	22.68 (21.01, 24.44)	23.03 (21.22, 24.98)	<0.001	22.94 (21.19, 24.97)	<0.001
Ethnic groups			<0.001		<0.001
Han	11,159 (83)	6476 (79)		2652 (84)	
Other minorities	2299 (17)	1692 (21)		520 (16)	
Education			<0.001		<0.001
None or lower than junior high school	6871 (51)	5350 (65)		1888 (60)	
Junior high school qualification	4690 (35)	2203 (27)		1019 (32)	
Senior high school qualification and higher	1897 (14)	615 (8)		265 (8)	
Smoking statues			<0.001		0.789
Non-smoker	9946 (74)	5815 (71)		2209 (70)	
Smoker	3512 (26)	2353 (29)		963 (30)	
Alcohol abuse			0.006		0.185
Non-alcohol abuser	11,410 (85)	6810 (83)		2517 (79)	
Alcohol abuser	2048 (15)	1358 (17)		655 (21)	
History of hypertension			<0.001		0.006
No	7139 (53)	1915 (23)		637 (20)	
Yes	2660 (20)	4046 (50)		1536 (48)	
Unknown	3659 (27)	2207 (27)		999 (31)	
History of diabetes			<0.001		<0.001
No	7943 (59)	4283 (52)		1603 (51)	
Yes	591 (4)	1019 (12)		217 (7)	
Unknown	4924 (37)	2866 (35)		1352 (43)	
History of atrial fibrillation			<0.001		0.028
No	11,289 (84)	5805 (71)		2193 (69)	
Yes	202 (2)	400 (5)		145 (5)	
Unknown	1967 (15)	1963 (24)		834 (26)	
History of coronary heart disease					
No	11,226 (83)	5533 (68)	<0.001	2218 (70)	0.015
Yes	498 (4)	990 (12)		262 (8)	
Unknown	1734 (13)	1645 (20)		692 (22)	
History of hyperlipidemia			<0.001		<0.001
No	5738 (43)	2573 (32)		894 (28)	
Yes	947 (7)	1604 (20)		532 (17)	
Unknown	6773 (50)	3991 (48)		1746 (55)	
Snore			<0.001		0.016
No	9175 (68)	4083 (50)		1472 (46)	
Yes	4283 (32)	4085 (50)		1700 (54)	

Values are medians (Q1, Q3) for consecutive characteristics. The proportion of data described in *n* (%). ^a^ P_1_: Comparison between ischemic stroke group and control group by chi-square test for categorical data and Mann–Whitney U test for continuous data. ^b^ P_2_: Comparison between intracerebral hemorrhage group and control group by chi-square test for categorical data and Mann–Whitney U test for continuous data.

**Table 2:** Stroke risk associated with sleep onset time and duration.

	Unadjusted Model		Model 1		Model 2	
	OR (95%CI)	*p* Value	OR (95%CI)	*p* Value	OR (95%CI)	*p* Value
Sleep onset time						
Ischemic stroke						
Before 10 p.m.	Ref	Ref	Ref	Ref	Ref	Ref
10–11 p.m.	0.623 (0.582,0.667)	<0.001	0.751 (0.700,0.807)	<0.001	0.764 (0.707,0.826)	<0.001
11 p.m.–12 a.m.	0.443 (0.380,0.518)	<0.001	0.774 (0.655,0.915)	0.003	0.677 (0.563,0.815)	<0.001
After 12 a.m.	1.045 (0.756,1.444)	0.792	1.499 (1.054,2.131)	0.024	1.297 (0.879,1.916)	0.190
Intracerebral hemorrhage						
Before 10 p.m.	Ref	Ref	Ref	Ref	Ref	Ref
10–11 p.m.	0.637 (0.578,0.702)	<0.001	0.684 (0.619,0.756)	<0.001	0.682 (0.612,0.760)	<0.001
11 p.m.–12 a.m.	0.567 (0.461,0.698)	<0.001	0.759 (0.611,0.943)	0.013	0.658 (0.520,0.832)	<0.001
After 12 a.m.	1.063 (0.680,1.661)	0.788	1.287 (0.811,2.043)	0.284	1.247 (0.752,2.068)	0.393
Sleep duration						
Ischemic stroke						
<6 h	Ref	Ref	Ref	Ref	Ref	Ref
6~8 h	0.348 (0.308,0.393)	<0.001	0.425 (0.376,0.482)	<0.001	0.494 (0.432,0.565)	<0.001
>8 h	0.471 (0.412,0.539)	<0.001	0.546 (0.476,0.627)	<0.001	0.604 (0.520,0.701)	<0.001
Intracerebral hemorrhage						
<6 h	Ref	Ref	Ref	Ref	Ref	Ref
6~8 h	0.475 (0.401,0.563)	<0.001	0.524 (0.441,0.622)	<0.001	0.607 (0.502,0.733)	<0.001
>8 h	0.595 (0.493,0.718)	<0.001	0.646 (0.534,0.781)	<0.001	0.733 (0.595,0.904)	0.004

Model 1: Adjusted age, gender, and BMI. Model 2: Adjusted age, gender, BMI, education, ethnicity, marriage, smoke history, drinking history, hypertension, diabetes, CVD, atrial fibrillation, and snore.

## Data Availability

The datasets generated and analyzed during this study are not publicly available due to privacy restrictions but may be available from the corresponding author on reasonable request and with permission from the institutional ethics committee.

## Supplementary Materials

The following supporting information can be downloaded at https://www.mdpi.com/article/10.3390/jcm14186529/s1, Figure S1: Subgroup analysis of effect of sleep time on ischemic stroke; Figure S2: Subgroup analysis of effect of sleep duration on ischemic stroke; Figure S3: Subgroup analysis of effect of sleep time on intracerebral hemorrhage; Figure S4: Subgroup analysis of effect of sleep duration on intracerebral hemorrhage; Figure S5: Sensitivity Analysis of Sleep Pattern Effects on Ischemic Stroke Risk in Male Participants; Figure S6: Sensitivity Analysis of Sleep Pattern Effects on Intracerebral hemorrhage Risk in Male Participants; Table S1: The sleep pattern survey.

## Abbreviations

The following abbreviations are used in this manuscript:

SOT	Sleep onset time
IS	Ischemic stroke
ICH	Intracerebral hemorrhage
