# Supplementary material for: The Influence of Optimal Sleep Onset Time and Duration on Risk of Stroke: A Community-Based, Cross-Sectional Study
Source: J Clin Med. 2025 Sep 17;14(18):6529. doi: 10.3390/jcm14186529 (PMC12470270; doi:10.3390/jcm14186529)
Supplement: Supplementary file 1 [file jcm-14-06529-s001.zip › jcm-3722494-supplementary.pdf]

Table S1. The sleep pattern survey

level	How many hours have you slept most nights for the past six months?	What time have you fallen asleep most nights in the past six months?
1	4 hours or less	before 10 p.m.
2	5 hours	between 10-11 p.m.
3	6 hours	between 11-12 p.m.
4	7 hours	after 12 p.m.
5	8 hours	
6	9 hours	
7	10 hours or more	

Figure S1. Subgroup analysis of effect of sleep time on ischemic stroke

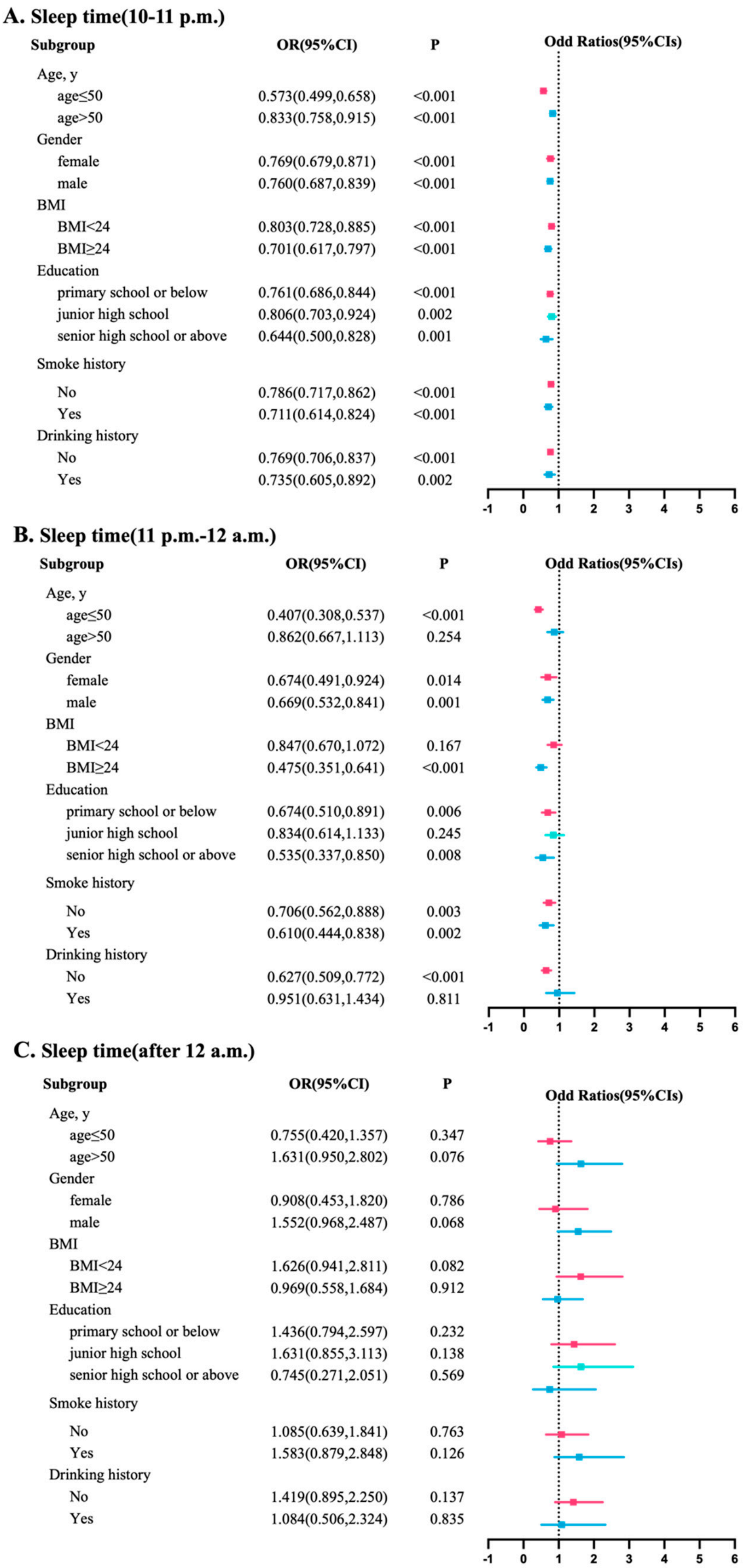

(A) Subgroup analysis of effect of sleep between 10 - 11 p.m. on ischemic stroke; (B) Subgroup analysis of effect of

sleep between 11 p.m. – 12 a.m. on ischemic stroke; (C) Subgroup analysis of effect of sleep after 12 a.m. on ischemic stroke.

Figure S2. Subgroup analysis of effect of sleep duration on ischemic stroke

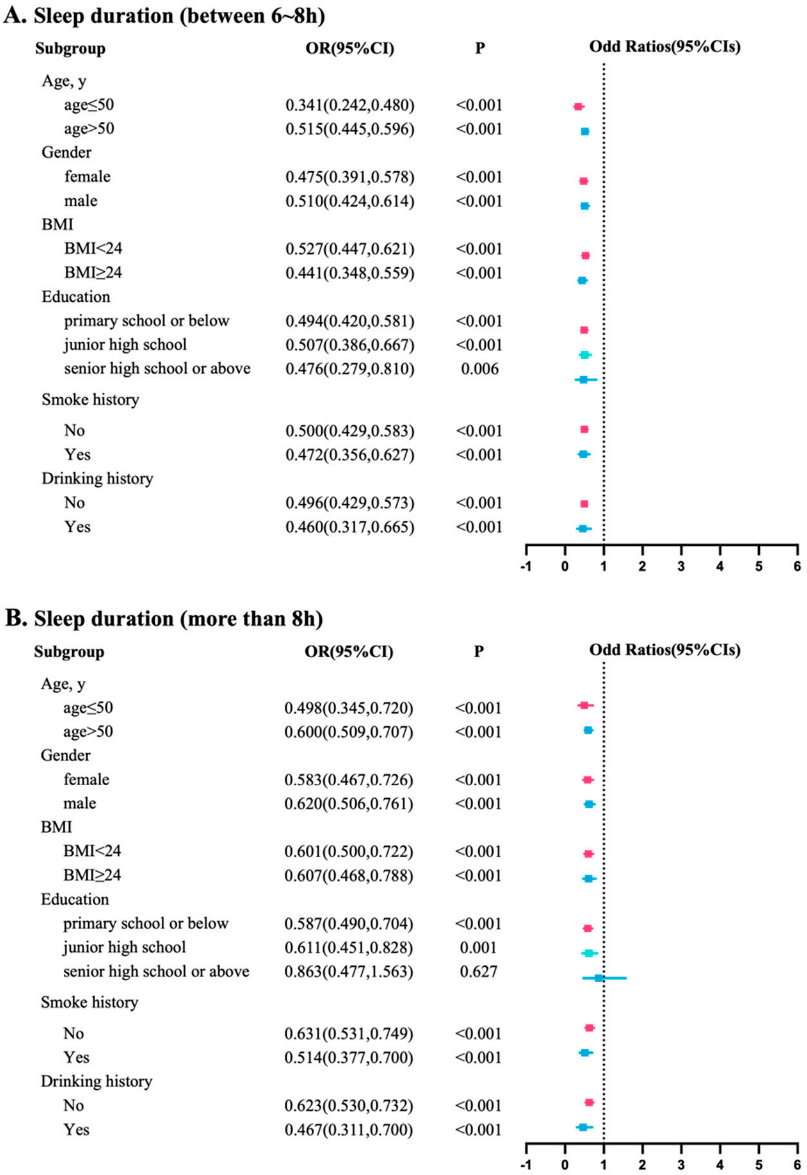

(A) Subgroup analysis of effect of sleep between 6 - 8 on ischemic stroke; (B) Subgroup analysis of effect of sleep more than 8h on ischemic stroke.

Figure S3. Subgroup analysis of effect of sleep time on intracerebral hemorrhage

A. Sleep time(10 - 11 p.m.)

Subgroup	OR(95%CI)	P
Age, y		
age≤50	0.476(0.331,0.683)	<0.001
age>50	0.730(0.652,0.817)	<0.001
Gender		
female	0.746(0.624,0.892)	0.001
male	0.650(0.567,0.745)	<0.001
BMI		
BMI<24	0.658(0.573,0.755)	<0.001
BMI≥24	0.724(0.597,0.879)	0.001
Education		
primary school or below	0.746(0.643,0.866)	<0.001
junior high school	0.686(0.574,0.820)	<0.001
senior high school or above	0.451(0.316,0.643)	<0.001
Smoke history		
No	0.689(0.605,0.784)	<0.001
Yes	0.683(0.560,0.833)	<0.001
Drinking history		
No	0.666(0.590,0.752)	<0.001
Yes	0.749(0.588,0.954)	0.019

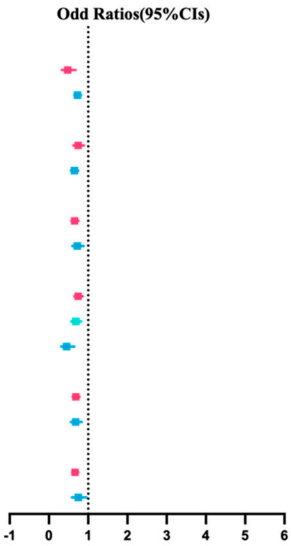

B. Sleep time(11 p.m. -12 a.m.)

Subgroup	OR(95%CI)	P
Age, y		
age≤50	0.570(0.334,0.973)	0.039
age>50	0.699(0.535,0.913)	0.009
Gender		
female	0.471(0.286,0.776)	0.003
male	0.718(0.547,0.942)	0.017
BMI		
BMI<24	0.576(0.414,0.801)	0.001
BMI≥24	0.742(0.506,1.087)	0.125
Education		
primary school or below	0.762(0.527,1.103)	0.15
junior high school	0.624(0.424,0.919)	0.017
senior high school or above	0.619(0.358,1.070)	0.086
Smoke history		
No	0.414(0.293,0.585)	<0.001
Yes	1.071(0.758,1.514)	0.698
Drinking history		
No	0.551(0.417,0.728)	<0.001
Yes	1.121(0.697,1.801)	0.638

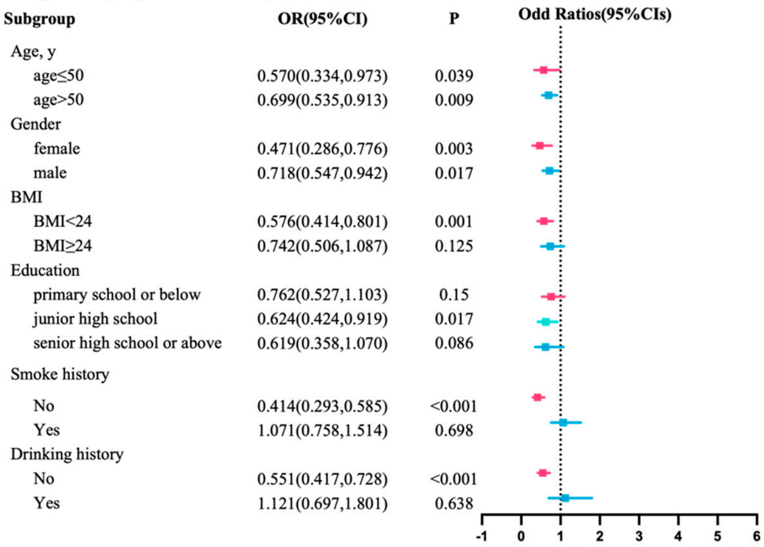

C. Sleep time(after 12 a.m.)

Subgroup	OR(95%CI)	P
Age, y		
age≤50	1.735(0.641,4.695)	0.278
age>50	1.111(0.615,2.007)	0.727
Gender		
female	1.411(0.597,3.331)	0.433
male	1.134(0.603,2.130)	0.696
BMI		
BMI<24	1.596(0.780,3.264)	0.201
BMI≥24	1.116(0.479,2.602)	0.799
Education		
primary school or below	1.034(0.415,2.579)	0.943
junior high school	1.140(0.502,2.587)	0.754
senior high school or above	2.198(0.834,5.791)	0.111
Smoke history		
No	1.154(0.567,2.347)	0.693
Yes	1.384(0.662,2.892)	0.388
Drinking history		
No	1.092(0.578,2.061)	0.786
Yes	1.643(0.686,3.930)	0.265

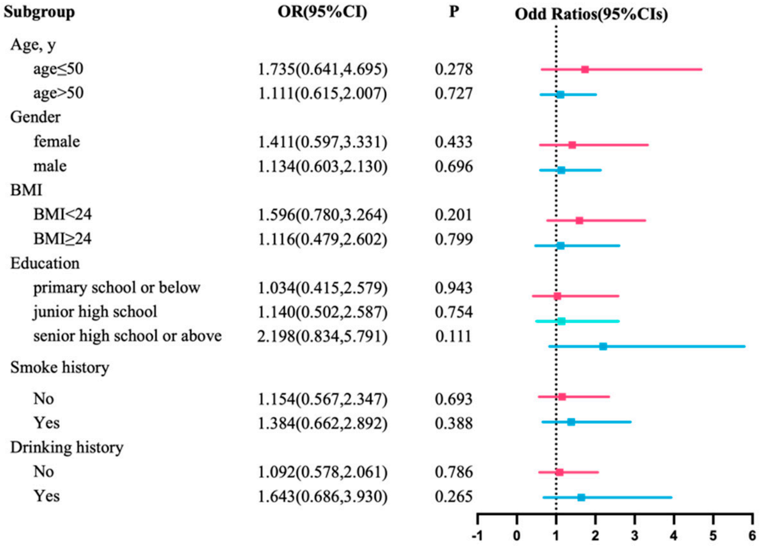

(A) Subgroup analysis of effect of sleep between 10 - 11 p.m. on intracerebral hemorrhage; (B) Subgroup analysis of effect of sleep between 11 p.m. – 12 a.m. on ischemic stroke; (C) Subgroup analysis of effect of sleep after 12 a.m. on ischemic stroke.

Figure S4. Subgroup analysis of effect of sleep duration on intracerebral hemorrhage

A. Sleep duration (6-8h)

Subgroup	OR(95%CI)	P
Age, y		
age≤50	0.298(0.103,0.862)	0.025
age>50	0.634(0.524,0.768)	<0.001
Gender		
female	0.547(0.413,0.725)	<0.001
male	0.651(0.504,0.843)	0.001
BMI		
BMI<24	0.710(0.560,0.900)	0.005
BMI≥24	0.461(0.323,0.657)	<0.001
Education		
primary school or below	0.622(0.492,0.785)	<0.001
junior high school	0.557(0.387,0.803)	0.002
senior high school or above	0.823(0.358,1.895)	0.647
Smoke history		
No	0.646(0.518,0.806)	<0.001
Yes	0.491(0.337,0.716)	<0.001
Drinking history		
No	0.641(0.520,0.789)	<0.001
Yes	0.429(0.266,0.691)	0.001

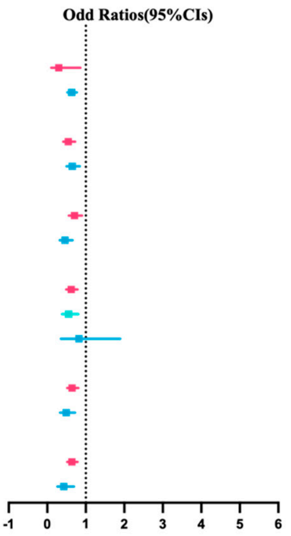

B. Sleep duration (>8h)

Subgroup	OR(95%CI)	P
Age, y		
age≤50	0.488(0.158,1.508)	0.213
age>50	0.746(0.603,0.923)	0.007
Gender		
female	0.755(0.552,1.033)	0.079
male	0.728(0.548,0.966)	0.028
BMI		
BMI<24	0.86(0.662,1.117)	0.259
BMI≥24	0.525(0.353,0.78)	0.001
Education		
primary school or below	0.746(0.576,0.965)	0.026
junior high school	0.637(0.425,0.955)	0.029
senior high school or above	1.513(0.612,3.737)	0.37
Smoke history		
No	0.858(0.672,1.095)	0.218
Yes	0.46(0.302,0.699)	<0.001
Drinking history		
No	0.81(0.643,1.02)	0.073
Yes	0.43(0.255,0.724)	0.002

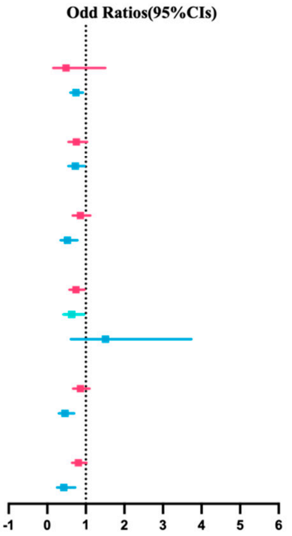

(A) Subgroup analysis of effect of sleep between 6 - 8 on intracerebral hemorrhage; (B) Subgroup analysis of effect of sleep more than 8h on intracerebral hemorrhage.

Figure S5. Sensitivity Analysis of Sleep Pattern Effects on Ischemic Stroke Risk in Male Participants

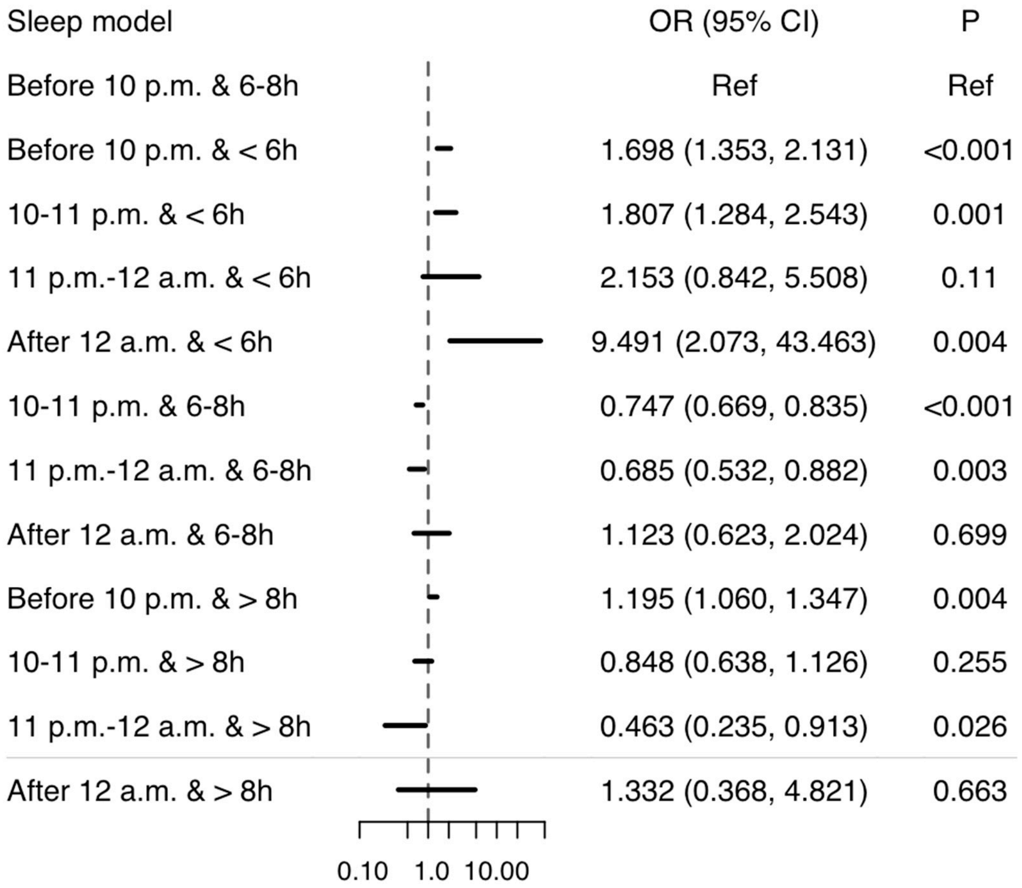

Adjusted odds ratios (ORs) with 95% confidence intervals for ischemic stroke risk associated with joint sleep duration/sleep onset time patterns in males (ischemic stroke: n =4,815, controls: n =7,534). Reference group: sleep onset before 10 p.m. with 6-8 hours duration.

Figure S6. Sensitivity Analysis of Sleep Pattern Effects on Intracerebral hemorrhage Risk in Male Participants

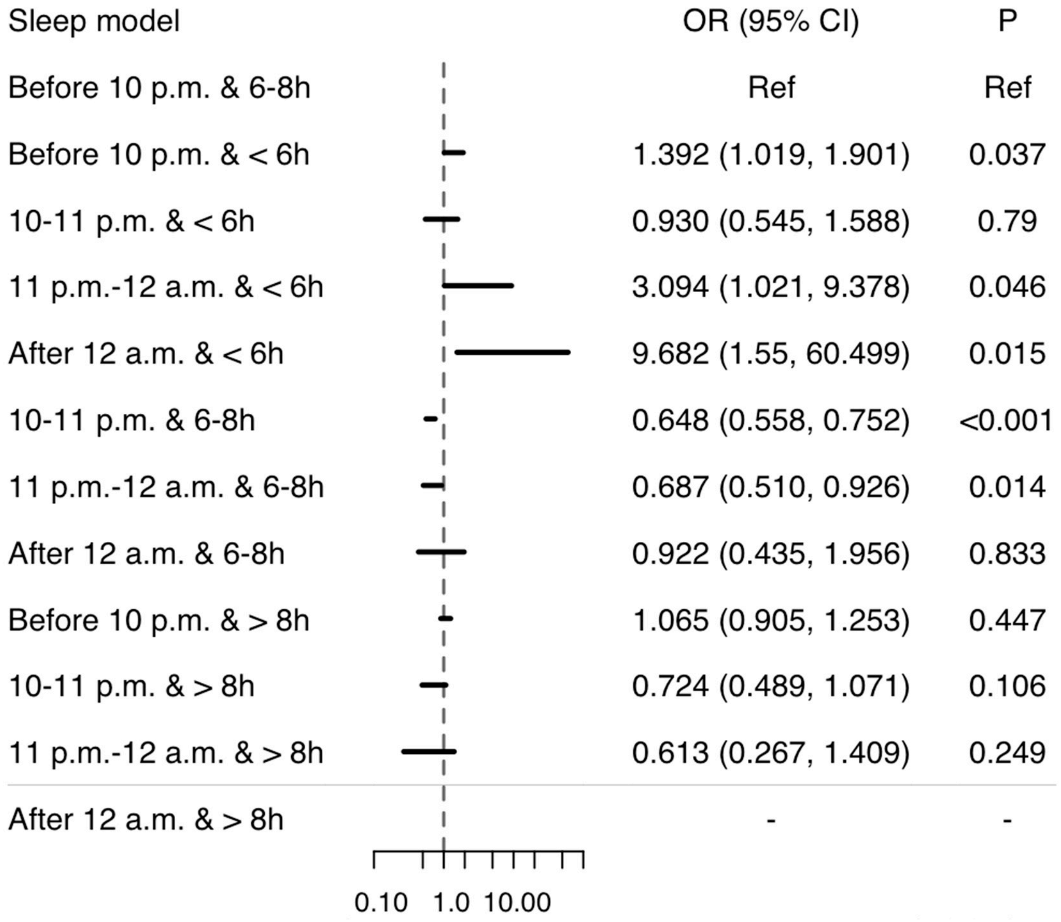

Adjusted odds ratios (ORs) with 95% confidence intervals for intracerebral hemorrhage risk associated with joint sleep duration/sleep onset time patterns in males (intracerebral hemorrhage: n =1,989, controls: n =7,534). Reference group: sleep onset before 10 p.m. with 6-8 hours duration.
